# Supplementary material for: Online Parallel Accumulation–Serial Fragmentation (PASEF) with a Novel Trapped Ion Mobility Mass Spectrometer
Source: Mol Cell Proteomics. 2018 Nov 1;17(12):2534–45. doi: 10.1074/mcp.TIR118.000900 (PMC6283298; doi:10.1074/mcp.TIR118.000900)
Supplement: supplemental Fig. S1 [file TIR118.000900_index.html]

Supplement to Online parallel accumulation – serial fragmentation (PASEF) with a novel trapped ion mobility mass spectrometer | Molecular & Cellular Proteomics

## Supplemental Data

- Suppl Figures 1 and 2 - Suppl Fig 1: PASEF precursor selection scheme Suppl Fig 2: Annotated MS/MS spectra of co-eluting and nearly isobaric peptide species
- Suppl Table 1 - Peptide Collisional Cross sections
