# Supplementary material for: Online Parallel Accumulation–Serial Fragmentation (PASEF) with a Novel Trapped Ion Mobility Mass Spectrometer
Source: Mol Cell Proteomics. 2018 Nov 1;17(12):2534–45. doi: 10.1074/mcp.TIR118.000900 (PMC6283298; doi:10.1074/mcp.TIR118.000900)
Supplement: supplemental Fig. S1 [file 138569_1_supp_227044_phgp3g.pdf]

Supplementary Figures for

Online parallel accumulation – serial fragmentation (PASEF) with a novel trapped ion mobility mass spectrometer

Florian Meier, Andreas-David Brunner, Scarlet Koch, Heiner Koch, Markus Lubeck, Michael Krause, Niels Goedecke, Jens Decker, Thomas Kosinski, Melvin A. Park, Nicolai Bache, Ole Hoerning, Jürgen Cox, Oliver Räther, Matthias Mann

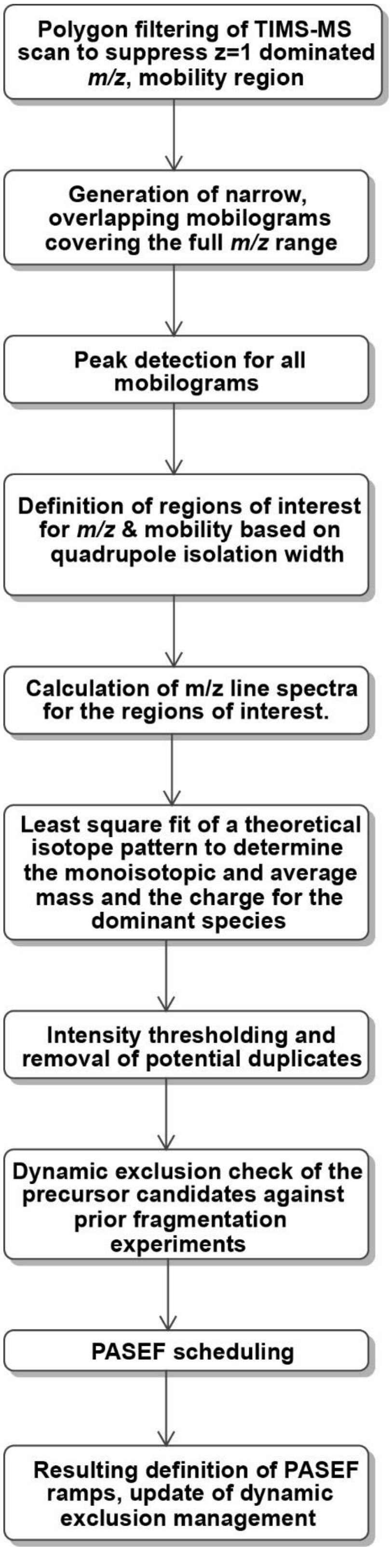

Supplementary Figure 1. Schematic of the online PASEF precursor ion selection algorithm.

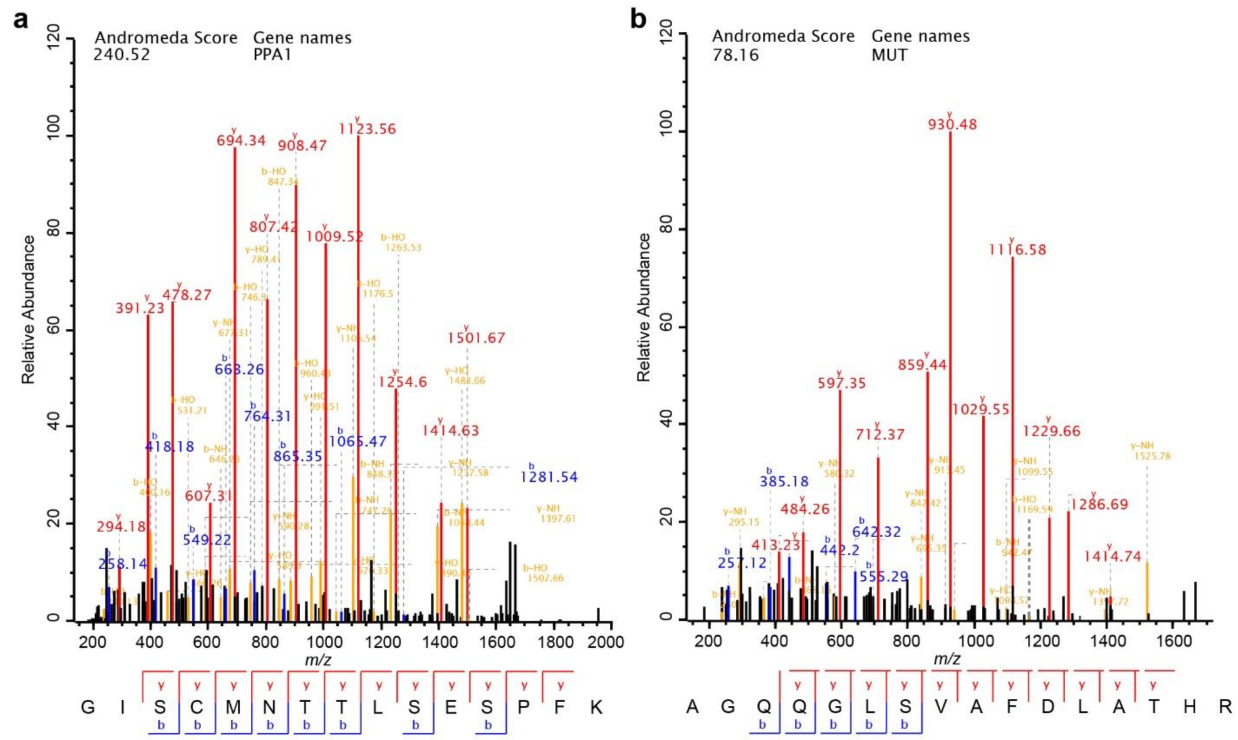

Supplementary Figure 2. MaxQuant identification of co-eluting peptides of very similar mass, which would have been co-fragmented without TIMS.
